# Autistic traits and obsessive–compulsive symptom severity: a retrospective six-month follow-up study

**DOI:** 10.3389/fpsyt.2026.1940901

**Published:** 2026-09-10

**Authors:** Yunus Akkeçili, Şebnem Pırıldar

**Affiliations:** 1 Department of Psychiatry, Dinar State Hospital, Afyonkarahisar, Türkiye; 2 Department of Psychiatry, Faculty of Medicine, Ege University, İzmir, Türkiye

**Keywords:** autistic traits, broader autism phenotype, compulsions, longitudinal study, obsessive–compulsive disorder

## Abstract

Autistic traits have been increasingly associated with obsessive–compulsive disorder (OCD), yet their influence on longitudinal symptom severity and treatment-related change remains poorly understood. This retrospective longitudinal study included 38 adults with DSM-5 OCD who completed six months of guideline-based pharmacological treatment. Obsessive–compulsive symptom severity was assessed using the Yale–Brown Obsessive–Compulsive Scale (Y-BOCS) at baseline, one month, and six months. Autistic traits were evaluated at the six-month follow-up using the Autism Spectrum Quotient (AQ). Linear mixed-effects models served as the primary analyses, while repeated-measures analyses of covariance were performed as sensitivity analyses. Exploratory analyses examined the contribution of individual AQ subscales. Higher autistic trait levels were independently associated with persistently greater OCD symptom severity across the six-month follow-up, whereas the rate of treatment-related improvement did not differ according to autistic trait levels. This association was primarily driven by compulsive rather than obsessive symptoms. Exploratory analyses further identified social skills and attention switching as the AQ dimensions most strongly associated with overall OCD severity and compulsive symptom burden, whereas attention to detail was not significantly associated with symptom severity. These findings suggest that autistic traits represent stable markers of greater clinical burden in OCD without necessarily conferring poorer pharmacological treatment responsiveness. The results further support a dimensional conceptualization of autistic traits and highlight social functioning as a potentially important contributor to OCD symptom burden.

## 1 Introduction

Obsessive–compulsive disorder (OCD) is a heterogeneous psychiatric condition characterized by obsessions and compulsions that are associated with substantial impairment in functioning and quality of life (1). Although evidence-based pharmacological and psychotherapeutic interventions are effective for many individuals with OCD, clinical outcomes vary considerably across patients (1). Traditional predictors such as baseline symptom severity, illness duration, and psychiatric comorbidity explain part of this variability; however, a substantial proportion of heterogeneity remains unexplained. This variability has stimulated increasing interest in dimensional neurodevelopmental characteristics that may influence the clinical expression and course of OCD (2, 3).

Autism spectrum disorder (ASD) is characterized by persistent differences in social communication and social reciprocity together with restricted and repetitive patterns of behavior and interests (4). Contemporary conceptualizations increasingly view autistic characteristics as dimensional rather than exclusively categorical phenomena. Within this framework, autistic traits are continuously distributed throughout the general population and can be observed at subclinical levels among individuals without a formal ASD diagnosis (5, 6). These traits encompass differences in social functioning, cognitive flexibility, communication style, and behavioral rigidity, all of which may influence vulnerability to and expression of psychiatric disorders (7, 8).

The relationship between OCD and ASD has attracted considerable attention because both conditions involve repetitive behaviors, resistance to change, and behavioral rigidity (9, 10). Rituals, sameness behaviors, and repetitive actions often appear phenomenologically similar across the two conditions, creating challenges for both conceptualization and differential diagnosis (9, 11). However, the functional properties of these behaviors differ substantially. In OCD, compulsions are typically performed to reduce distress, neutralize intrusive thoughts, or prevent feared consequences (1, 12). In contrast, repetitive behaviors in ASD are more often associated with sensory regulation, predictability, self-regulation, or intrinsically rewarding routines (12, 13). Qualitative evidence further suggests that autism-related repetitive behaviors are more frequently experienced as ego-syntonic, whereas OCD compulsions are generally ego-dystonic and associated with distress and anxiety (14, 15). Despite these functional differences, the overlap between repetitive behavioral dimensions suggests that shared neurocognitive mechanisms may contribute to symptom expression across both conditions.

Epidemiological evidence further supports a meaningful relationship between OCD and autism-related characteristics. Longitudinal population-based studies have demonstrated a bidirectional association between OCD and ASD, indicating that individuals with one condition are at increased risk of developing the other (16). Recent reviews and meta-analytic evidence similarly suggest elevated rates of OCD among autistic individuals and increased prevalence of ASD characteristics within OCD populations (9, 17). Moreover, the co-occurrence of autistic characteristics and OCD has been associated with greater psychosocial impairment, poorer quality of life, and increased clinical complexity (2, 8).

Beyond categorical diagnoses, growing evidence suggests that autistic traits function as transdiagnostic modifiers across psychiatric disorders. Elevated autistic traits have been associated with greater symptom burden and poorer functioning in anxiety disorders and related internalizing conditions (7, 8). Within OCD specifically, several studies have reported positive associations between autistic traits and symptom severity. Dell’Osso et al. (18) found that autistic trait dimensions, particularly inflexibility and communication difficulties, were associated with increased OCD-spectrum severity. Similarly, Doi et al. (19) reported that higher autistic trait levels were related to greater obsessive–compulsive symptom severity and lower psychological well-being among adults with OCD. In a UK outpatient sample, elevated autistic traits were associated with increased OCD severity and poorer insight (20). More recently, Tumkaya et al. (21) demonstrated that autistic traits, particularly difficulties in attention switching, predicted obsessive–compulsive symptom burden even after adjustment for demographic and affective variables. Collectively, these findings suggest that autistic traits contribute meaningfully to the clinical expression of OCD, although the mechanisms underlying this association remain incompletely understood.

One plausible mechanism linking autistic traits to OCD severity is cognitive inflexibility. Cognitive flexibility refers to the capacity to modify behavior in response to changing environmental demands and to shift attention between competing cognitive sets. Comparative reviews of ASD and OCD have identified cognitive rigidity, executive dysfunction, and anxiety-related processes as shared mechanisms underlying repetitive behavior expression (22). Experimental studies have demonstrated reduced behavioral flexibility and increased perseverative responding among individuals with ASD, with these impairments closely related to repetitive behavioral tendencies (23). Consistent with these findings, a recent meta-analysis reported robust deficits in cognitive flexibility across autism spectrum populations (24). Given that compulsive behaviors in OCD are similarly characterized by rigidity and difficulty disengaging from repetitive actions, cognitive inflexibility may represent a key mechanism linking autistic traits and obsessive–compulsive symptom severity.

Social-cognitive processes may provide an additional explanatory pathway. Difficulties in social reciprocity constitute a core dimension of autistic traits, and growing evidence suggests that atypical predictive processing contributes to these impairments. Neuroimaging studies have demonstrated altered social prediction-error signaling in ASD, with the magnitude of these abnormalities correlating with measures of social dysfunction (25). Difficulties in social prediction and uncertainty processing may increase vulnerability to anxiety and maladaptive coping responses, particularly in situations characterized by ambiguity and reduced predictability. Because intolerance of uncertainty has also been implicated in OCD, autistic-trait dimensions related to social functioning may contribute to the development and maintenance of obsessive–compulsive symptoms through overlapping cognitive mechanisms.

Despite growing evidence linking autistic traits to OCD severity, important gaps remain. Most existing studies have relied on cross-sectional designs, limiting conclusions regarding the longitudinal impact of autistic traits on symptom trajectories. Existing longitudinal evidence suggests that elevated autistic traits are associated with greater psychosocial burden and functional impairment among individuals with OCD; however, their influence on treatment outcomes remains less clear (2). Consequently, it remains uncertain whether autistic traits merely reflect greater baseline symptom burden or whether they also influence symptom improvement over time. Longitudinal evidence from related anxiety disorders is similarly limited. In a six-month follow-up study of panic disorder, autistic traits were associated with greater symptom severity across repeated assessments but did not significantly alter the trajectory of treatment-related improvement during pharmacological treatment (26). A broadly similar pattern may be present in OCD, but direct evidence remains sparse and inconclusive (2).

Accordingly, the present study investigated the association between autistic traits and both symptom severity and longitudinal symptom trajectories in adults with OCD receiving standard pharmacological treatment. Using repeated assessments over a six-month follow-up period, we examined the extent to which autistic traits were associated with overall obsessive–compulsive symptom burden and whether they influenced rates of symptom improvement over time. Based on previous cross-sectional findings and emerging longitudinal evidence, we hypothesized that higher levels of autistic traits would be associated with greater OCD severity, particularly within symptom dimensions characterized by behavioral rigidity, while exerting limited influence on treatment-related symptom trajectories.

## 2 Methods

### 2.1 Study design and participants

This retrospective observational cohort study was conducted in the Psychiatry Outpatient Clinic of Dinar State Hospital, Türkiye. Between January 2025 and July 2025, consecutive adult patients who had completed at least six months of routine outpatient follow-up for obsessive–compulsive disorder (OCD) were screened for eligibility. Patients were recruited consecutively to minimize selection bias. Since 2023, all patients with OCD attending the clinic have been routinely monitored using the Yale–Brown Obsessive–Compulsive Scale (Y-BOCS) at baseline and follow-up visits as part of standard clinical care, with scores systematically documented in the medical records. All Y-BOCS assessments were performed by the same psychiatrist throughout the follow-up period. Clinical outcome data collected during routine care were retrospectively extracted from these records, whereas autistic traits were assessed cross-sectionally by administering the Autism Spectrum Quotient (AQ) once, at or after the scheduled six-month follow-up visit following written informed consent.

OCD diagnoses were established by an experienced psychiatrist according to the *Diagnostic and Statistical Manual of Mental Disorders, Fifth Edition* (DSM-5) diagnostic criteria during routine clinical evaluation. The Y-BOCS was subsequently administered to quantify symptom severity and was not used as a diagnostic instrument. Owing to the retrospective naturalistic design of the study, structured diagnostic interviews (e.g., SCID-5) were not performed. Likewise, psychiatric comorbidities were evaluated clinically during routine care but were not systematically assessed using standardized diagnostic interviews.

All participants received guideline-based pharmacological treatment for OCD, consisting primarily of selective serotonin reuptake inhibitor (SSRI)-based regimens. When clinically indicated, augmentation strategies with low-dose atypical antipsychotics were implemented according to contemporary treatment guidelines. The most frequently prescribed regimen was escitalopram monotherapy, followed by escitalopram augmented with aripiprazole and, less frequently, clomipramine-based combinations. The pharmacological regimens maintained during the six-month follow-up are presented in **Supplementary Table 1**. Previous treatment history, including prior pharmacotherapy and cognitive-behavioral therapy/exposure and response prevention (CBT/ERP), was extracted from the clinical records and is summarized in **Supplementary Table 2**.

Eligible participants were adults aged 18–65 years with a DSM-5 diagnosis of OCD who had completed at least six consecutive months of pharmacological treatment and follow-up at the same outpatient clinic. To reduce treatment-related heterogeneity, individuals who had received additional psychiatric treatment or structured psychotherapy at another psychiatric center during the preceding year were excluded. Additional exclusion criteria were: (1) a current or lifetime diagnosis of psychotic disorder, bipolar disorder, or substance use disorder, and (2) significant cognitive impairment (e.g., dementia or intellectual disability) that could interfere with valid completion of self-report measures.

At the scheduled follow-up visit occurring at or after completion of the six-month follow-up period, eligible patients were invited to participate in the study. Those who provided written informed consent completed the Turkish version of the Autism Spectrum Quotient (AQ) according to the standard administration procedure without additional research-specific instructions. Participants were not instructed to rate their characteristics specifically according to childhood, premorbid functioning, or the period preceding OCD onset. Consequently, AQ responses may have reflected participants’ contemporaneous self-perception at the time of assessment. Consistent with previous psychiatric studies employing the AQ in adult psychiatric populations (2), AQ scores were conceptualized as dimensional trait measures.

The study focused on dimensional autistic traits rather than categorical autism spectrum disorder (ASD). None of the participants had a documented DSM-5 diagnosis of ASD at the time of assessment.

During the recruitment period, 45 patients met the initial eligibility criteria. Of these, 38 provided written informed consent and completed the AQ assessment. No participant withdrew after enrollment. The remaining eligible patients declined participation because of insufficient time during the outpatient visit, and no research data were collected from these individuals. Because inclusion required completion of the six-month follow-up, the present sample represents a treatment-completer cohort rather than an inception cohort of all patients initially diagnosed with OCD. Consequently, the findings should be interpreted within the context of treatment completers and may not generalize to individuals who discontinue treatment prematurely or are lost to follow-up.

Ethical approval was obtained from the Afyonkarahisar Health Sciences University Ethics Committee (Approval Date: December 13, 2024; Meeting No: 2024/11). All participants provided written informed consent before participation.

**Figure 1:**
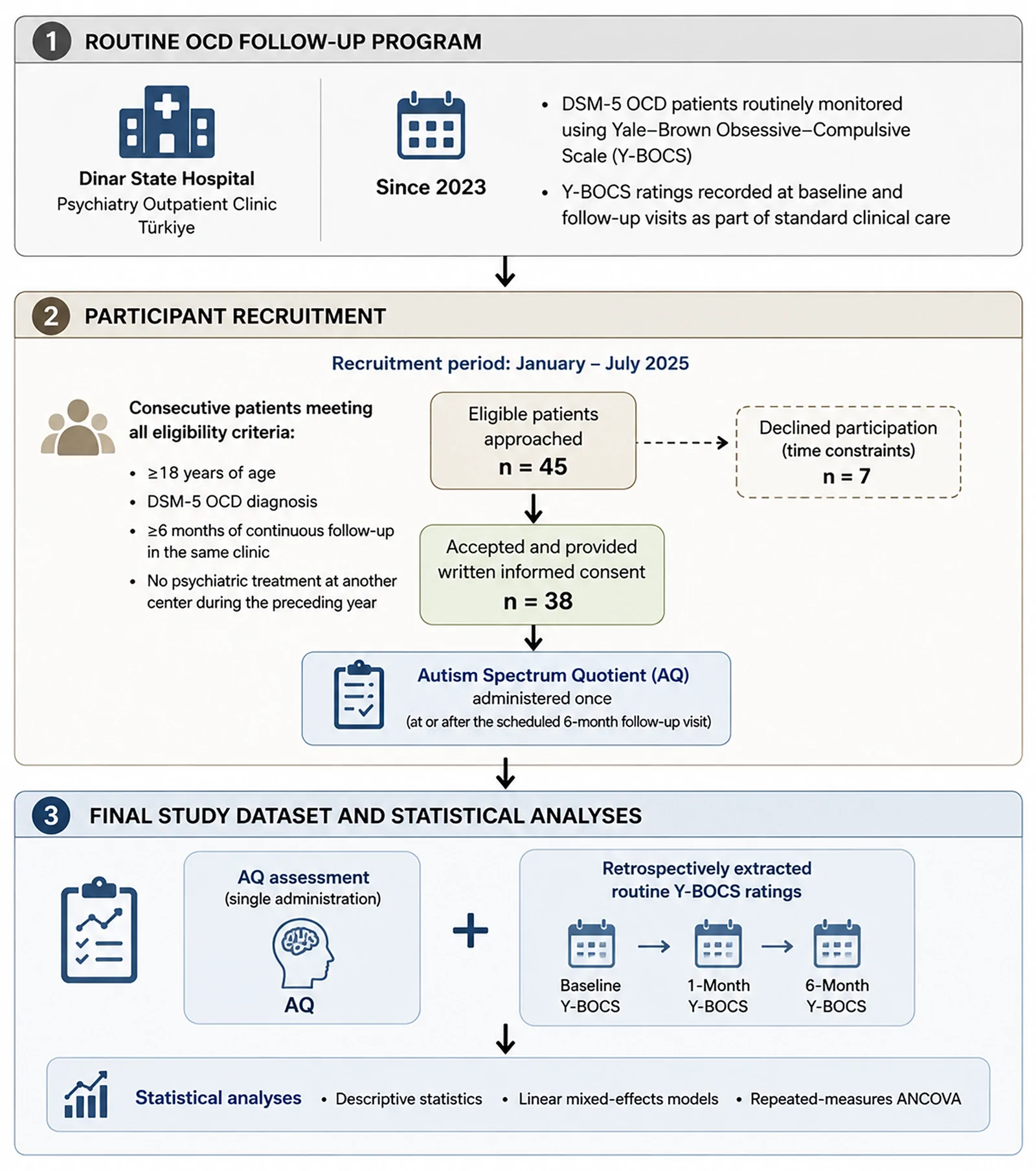
Study design, participant recruitment, and data collection workflow. AQ was administered once at or after the scheduled six-month follow-up visit. Y-BOCS scores were retrospectively extracted from routine clinical records. AQ, Autism Spectrum Quotient; Y-BOCS, Yale–Brown Obsessive–Compulsive Scale.

A total of 38 participants met the eligibility criteria and were included in the final analyses. Sociodemographic variables, including age and sex, were obtained from the clinical records. AQ total and subscale scores obtained at the study assessment visit (conducted at or after completion of the six-month follow-up period), together with Y-BOCS total and subscale scores retrospectively extracted from routine clinical records at baseline, one-month, and six-month follow-up assessments, were used to examine the relationship between autistic traits and OCD symptom severity over time. The study workflow is illustrated in **Figure 1**.

### 2.2 Measures

#### 2.2.1 Autism spectrum quotient

The Autism Spectrum Quotient (AQ) is a 50-item self-report questionnaire developed to assess autistic traits in adults with normal intellectual functioning. The instrument comprises five domains (Social Skills, Attention Switching, Attention to Detail, Communication, and Imagination) and was designed to quantify autistic characteristics across the broader autism phenotype rather than to establish a clinical diagnosis of autism spectrum disorder (27). The Turkish version has demonstrated acceptable reliability and construct validity (28). Consistent with the dimensional framework of the present study, AQ total and subscale scores were analyzed as continuous variables rather than categorized according to proposed screening cut-off values.

#### 2.2.2 Yale–Brown obsessive–compulsive scale

The Yale–Brown Obsessive–Compulsive Scale (Y-BOCS) is a clinician-administered instrument widely regarded as the gold standard measure of obsessive–compulsive symptom severity. The scale consists of 10 items rated on a 5-point Likert scale (0–4), yielding a total score ranging from 0 to 40. Separate subscale scores are calculated for obsessive and compulsive symptom severity.

The Turkish version of the Y-BOCS has demonstrated satisfactory reliability and validity in clinical populations (29).

### 2.3 Statistical analysis

All statistical analyses were conducted using IBM SPSS Statistics Version 25.0. Descriptive statistics were calculated for demographic and clinical variables. Continuous variables were evaluated for normality using visual inspection of histograms and Q–Q plots.

Linear mixed-effects models (LMMs) were employed as the primary analytical framework because they allow efficient modeling of repeated observations while accounting for within-subject correlations. Separate models were estimated for Y-BOCS total, obsession, and compulsion scores. Time (baseline, one month, and six months), AQ total score, age, and sex were entered as fixed effects, and the interaction between time and AQ total score was included to evaluate whether autistic traits influenced symptom trajectories over time.

Participant identification number was specified as a random intercept to account for individual-level variability. Repeated measurements were modeled using an unstructured covariance matrix, allowing the covariance among assessment time points to be estimated directly from the data. Models were estimated using maximum likelihood (ML). Model fit was evaluated using Akaike’s Information Criterion (AIC) and Bayesian Information Criterion (BIC).

To evaluate the robustness of the primary findings, repeated-measures analyses of covariance (RM-ANCOVA) were conducted as secondary sensitivity analyses. In these models, Y-BOCS total, obsession, and compulsion scores were specified as within-subject variables, whereas AQ total scores were entered as continuous covariates. Age and sex were included as covariates in all analyses to account for potential demographic confounding.

To further explore the dimensions of autistic traits associated with OCD symptom severity, separate exploratory RM-ANCOVA models were conducted using each AQ subscale (Social Skills, Attention Switching, Attention to Detail, Communication, and Imagination). Subscales were examined individually to avoid multicollinearity and to identify the dimensions primarily responsible for observed AQ total score effects.

For RM-ANCOVA analyses, sphericity was assessed using Mauchly’s test. When the assumption of sphericity was violated, Greenhouse–Geisser corrections were applied. Effect sizes were reported as partial eta-squared (η²p), with values of.01,.06, and.14 representing small, medium, and large effects, respectively.

All statistical tests were two-tailed, and statistical significance was defined as p <.05. Because AQ subscale analyses were exploratory in nature, their results were interpreted cautiously and viewed primarily as hypothesis-generating rather than confirmatory. Given the modest sample size, particular caution was exercised when interpreting non-significant findings and interaction effects.

## 3 Results

During the recruitment period, 45 patients met the initial eligibility criteria. Of these, 38 provided written informed consent and completed the Autism Spectrum Quotient (AQ) assessment, constituting the final study sample. The remaining eligible patients declined participation because of insufficient time during the outpatient visit.

The final sample consisted of 38 adults with a clinical DSM-5 diagnosis of obsessive–compulsive disorder. The mean age was 37.92 years (SD = 13.00), and 26 participants (68.4%) were female. The mean AQ total score for the entire sample was 24.03 (SD = 5.90), indicating a broad dimensional distribution of autistic traits within this clinical OCD cohort.

Although females constituted approximately two-thirds of the sample, male participants had significantly higher AQ total scores than female participants (27.67 ± 6.10 vs. 22.35 ± 5.08). This difference remained significant after adjustment for age (ANCOVA: *F*(1, 35) = 7.16, *p* = .011, η²_p_ = .170), whereas age itself was not significantly associated with AQ scores (*F*(1, 35) = 0.70, *p* = .409). Detailed demographic and clinical characteristics are presented in **Table 1**.

**Table 1:** Demographic and clinical characteristics of the participants (N = 38).

Variable	n (%)	Mean (SD)
Demographic characteristics		
Age (years)	–	37.92 (13.00)
Sex		
• Male	12 (31.6)	–
• Female	26 (68.4)	–
Years of education	–	9.82 (3.53)
Family history of psychiatric disorders	18 (47.4)	–
Clinical characteristics		
Autism Spectrum Quotient (AQ), overall	–	24.03 (5.90)
• AQ (Male)	–	27.67 (6.10)
• AQ (Female)	–	22.35 (5.08)
AQ subscale scores		
Social Skills	–	5.37 (2.26)
Attention Switching	–	5.50 (1.94)
Attention to Detail	–	5.32 (1.82)
Communication	–	4.05 (2.11)
Imagination	–	3.79 (1.95)

Values are presented as n (%) for categorical variables and mean (SD) for continuous variables. Higher AQ scores indicate greater autistic trait burden. The Autism Spectrum Quotient (AQ) is a dimensional measure of autistic traits and is not intended as a diagnostic instrument for autism spectrum disorder. AQ, Autism Spectrum Quotient; SD, standard deviation.

**Figure 2:**
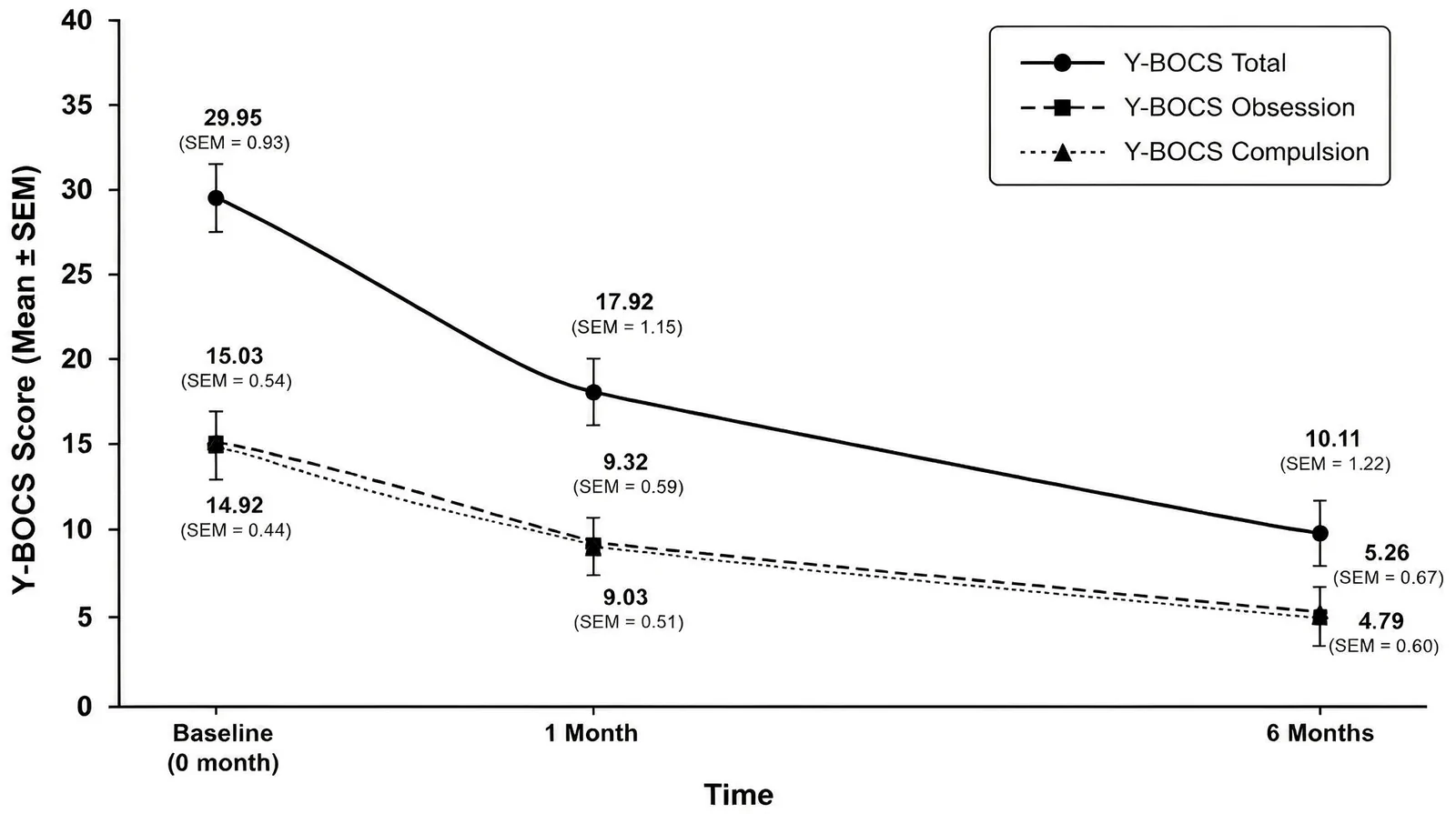
Longitudinal changes in obsessive–compulsive symptom severity. Mean Y-BOCS total, obsession, and compulsion scores are presented at baseline, 1-month, and 6-month assessments. Error bars represent the standard error of the mean (SEM).

Regarding clinical severity, Yale–Brown Obsessive–Compulsive Scale (Y-BOCS) total scores demonstrated a marked reduction across the six-month follow-up period, decreasing from baseline (M = 29.95, SD = 5.75) to one month (M = 17.92, SD = 7.09) and six months (M = 10.11, SD = 7.54). Similar improvement trajectories were observed for both obsession and compulsion subscale scores (**Figure 2**).

### 3.1 Linear mixed-effects models (primary analyses)

Linear mixed-effects models were conducted to examine the longitudinal association between autistic traits and obsessive–compulsive symptom severity while adjusting for age and sex.

For Y-BOCS total severity, a significant main effect of time was observed, F(2, 47.56) = 20.82, p <.001, indicating a robust reduction in symptom severity across the follow-up period. AQ total scores were significantly associated with overall OCD severity, F(1, 38.62) = 5.79, p = .021, with higher AQ scores corresponding to greater symptom burden. The time × AQ interaction was not significant (p = .742), suggesting that autistic traits did not influence the trajectory of symptom improvement. Neither age (p = .315) nor sex (p = .921) emerged as significant predictors.

Analyses of the Y-BOCS subscales revealed differential associations between autistic traits and obsessive versus compulsive symptom severity. For obsessive symptoms, a significant effect of time was observed, F(2, 46.91) = 22.04, p <.001; however, AQ total scores were not significantly associated with obsessive severity (p = .119), and the time × AQ interaction was not significant (p = .715).

In contrast, for compulsive symptoms, both a significant main effect of time, F(2, 47.83) = 16.71, p <.001, and a significant association with AQ total scores were observed, F(1, 38.60) = 8.61, p = .006. Higher levels of autistic traits were associated with greater compulsive symptom severity across all time points. The time × AQ interaction remained non-significant (p = .690), indicating that autistic traits did not influence the rate of symptom improvement. Full model estimates and fit indices are presented in **Supplementary Table 3**.

### 3.2 Repeated-measures ANCOVA (sensitivity analyses)

Repeated-measures ANCOVA (RM-ANCOVA) analyses were conducted as sensitivity analyses to examine the robustness of the primary mixed-effects model findings while adjusting for age and sex.

For overall OCD severity (Y-BOCS total), a significant main effect of time was observed (F(2, 68) = 20.36, p <.001, η²p = .375), indicating a substantial reduction in symptom severity across the assessment period. After controlling for age and sex, AQ total scores remained significantly associated with overall symptom burden (F(1, 34) = 7.68, p = .009, η²p = .184). The time × AQ interaction was not significant (p = .216), suggesting that autistic traits did not influence the trajectory of symptom improvement.

RM-ANCOVA demonstrated the same differential pattern across OCD symptom dimensions, confirming that autistic traits were associated with compulsive but not obsessive symptom severity. For obsessive symptoms, AQ total scores were not significantly associated with symptom severity (p = .059), and no significant time × AQ interaction was observed (p >.05), consistent with the findings from mixed-effects models.

In contrast, for compulsive symptoms, RM-ANCOVA confirmed a significant association between AQ total scores and symptom severity (F(1, 34) = 9.09, p = .005, η²p = .211), alongside a significant main effect of time (F(2, 68) = 18.18, p <.001, η²p = .348). The time × AQ interaction was not significant (p = .233), indicating that autistic traits were associated with greater compulsive symptom burden but did not influence the rate of symptom improvement.

Across all models, age and sex did not emerge as significant predictors, and their inclusion did not alter the observed associations between autistic traits and symptom severity.

### 3.3 Exploratory analyses of AQ subscales

Exploratory analyses were conducted to identify specific dimensions of autistic traits associated with OCD symptom severity.

For overall OCD severity, two AQ subscales emerged as significant predictors. The social skills subscale demonstrated a strong association with symptom severity (F(1, 34) = 12.75, p = .001, η²p = .273), representing the largest observed effect size among all subscales. Similarly, the attention switching subscale was significantly associated with OCD severity (F(1, 34) = 9.34, p = .004, η²p = .215), indicating that reduced cognitive flexibility was also linked to greater symptom burden.

In contrast, the attention to detail (p = .920), communication (p = .125), and imagination (p = .447) subscales were not significantly associated with OCD severity, suggesting that the relationship between autistic traits and OCD is not generalized across all domains but is instead driven by specific dimensions related to social functioning and cognitive flexibility.

Exploratory analyses focusing on compulsive symptoms revealed an even more pronounced pattern. The social skills subscale remained the strongest predictor of compulsive severity (F(1, 34) = 11.55, p = .002, η²p = .254), followed by the attention switching subscale (F(1, 34) = 9.96, p = .003, η²p = .227). These findings indicate that deficits in social functioning and cognitive flexibility are specifically associated with increased compulsive symptom burden.

In contrast, the attention to detail (p = .939), communication (p = .105), and imagination (p = .878) subscales were not significantly associated with compulsive symptoms. This selective pattern supports the specificity of the observed associations and argues against a generalized effect of autistic traits across all domains.

Across all subscale models, no significant time × subscale interaction effects were observed (all p >.05), indicating that these dimensions did not influence the trajectory of symptom improvement.

Full model estimates and fit indices are presented in **Supplementary Table 4**.

## 4 Discussion

The present study examined the association between autistic traits and obsessive–compulsive symptom severity using a longitudinal design and complementary analytic approaches. Across both linear mixed-effects models and RM-ANCOVA analyses, autistic traits were significantly associated with greater overall OCD severity. However, this relationship was not uniform across symptom dimensions and appeared to be primarily driven by compulsive symptoms rather than obsessive symptomatology. Importantly, although symptom severity decreased substantially over time across the entire cohort, individuals with higher autistic trait levels consistently exhibited greater symptom burden throughout the six-month follow-up, and autistic traits were not associated with the rate of symptom improvement over time. Thus, autistic traits appeared to influence the overall level of symptom severity rather than the trajectory of treatment-related improvement. Together, these findings suggest that autistic traits may function as stable markers of clinical burden in OCD rather than modifiers of treatment response. This interpretation is broadly consistent with previous evidence showing that elevated autistic traits are associated with greater OCD severity, while available longitudinal findings suggest a less consistent association with treatment outcomes (2, 20). From a clinical perspective, these findings indicate that patients with elevated autistic traits may remain more symptomatic throughout treatment while still achieving improvements comparable in magnitude to those with lower autistic trait levels.

A central finding of the present study is the selective association between autistic traits and compulsive, rather than obsessive, symptom severity. Although previous studies have consistently reported positive associations between autistic traits and overall OCD severity (18–21), relatively little attention has been paid to whether this relationship differs across symptom dimensions. The present findings extend this literature by suggesting that autistic traits may be more closely linked to the compulsive component of OCD. This observation is consistent with contemporary conceptualizations emphasizing that repetitive behaviors constitute an important area of overlap between OCD and ASD, despite differing in their underlying function and motivational context (9, 12). Whereas compulsions in OCD are typically performed to reduce distress or prevent feared outcomes, repetitive behaviors in ASD are generally regarded as intrinsically rewarding or regulatory. The present findings suggest that autistic traits may be more closely related to the behavioral manifestations of OCD than to obsessive symptom severity. Nevertheless, given the limited symptom-dimension literature currently available, this interpretation should be considered preliminary and requires replication in larger prospective cohorts.

Exploratory analyses of AQ subscales further refined this pattern by identifying social skills and attention switching as the dimensions most strongly associated with OCD symptom severity. Contrary to our initial expectation, attention to detail, a characteristic often considered conceptually relevant to OCD, was not associated with symptom severity. Instead, deficits in social skills emerged as the strongest predictor of both overall OCD severity and compulsive symptom burden, while attention switching demonstrated an additional independent contribution. Communication, imagination, and attention to detail were not significantly associated with symptom severity. These findings suggest that the relationship between autistic traits and OCD is not generalized across all domains of the broader autism phenotype but may instead be driven by specific dimensions related to social functioning and cognitive flexibility.

The contribution of attention switching is consistent with previous studies implicating cognitive inflexibility in the association between autistic traits and OCD symptoms (20, 21). Comparative reviews have similarly proposed reduced cognitive flexibility as one of the principal mechanisms underlying repetitive behaviors across both ASD and OCD (22). Experimental studies have demonstrated impaired behavioral flexibility and increased perseverative responding in autism spectrum conditions (23), while meta-analytic evidence has confirmed robust deficits in cognitive flexibility across autistic populations (24). Although the present study did not directly assess executive functioning, the observed association between attention-switching difficulties and compulsive symptom severity is consistent with the hypothesis that reduced cognitive flexibility may contribute to the persistence of compulsive behaviors.

The findings related to social skills warrant particular attention. Although previous OCD studies have more consistently emphasized attention-switching difficulties and broader social dysfunction among individuals with elevated autistic traits (3, 20), social skills emerged as the strongest AQ dimension associated with symptom severity in the present sample. This finding extends previous work by suggesting that social functioning may represent an important correlate of symptom burden in at least a subgroup of patients with OCD. One possible explanation is that impairments in social reciprocity and interpersonal functioning contribute to greater psychosocial burden, thereby amplifying compulsive symptom expression. This interpretation is broadly consistent with social-cognitive models of autism spectrum conditions (25), although the present study did not directly examine these mechanisms. Accordingly, these findings should be regarded as hypothesis-generating and require confirmation in prospective studies incorporating comprehensive assessments of social cognition, interpersonal functioning, and executive processes.

The absence of a significant time × AQ interaction is particularly noteworthy. Our findings extend previous work by Mito et al. (2), who reported greater clinical burden among patients with elevated autistic traits, and are consistent with our previous longitudinal study in panic disorder (26), in which autistic traits were associated with greater symptom severity but not with differential treatment trajectories. Thus, autistic traits were associated with a persistently greater symptom burden rather than with a differential rate of symptom improvement.Together, these findings suggest that autistic traits may function primarily as stable markers of clinical burden rather than determinants of pharmacological treatment responsiveness. From a clinical perspective, this distinction is important because it indicates that patients with elevated autistic traits should not necessarily be expected to respond less favorably to standard pharmacological treatment despite presenting with greater symptom severity.

Although approximately two-thirds of the present sample were female, neither age nor sex emerged as significant predictors in the primary longitudinal models, and the associations between autistic traits and OCD symptom severity remained significant after adjustment for these demographic variables. Moreover, male participants demonstrated significantly higher AQ scores than females, consistent with previous studies of autistic traits. Nevertheless, sex did not account for the observed relationship between AQ scores and OCD severity. Replication in larger and more sex-balanced cohorts is warranted to further evaluate the generalizability of these findings.

Several limitations should be considered when interpreting the present findings. First, the sample size was relatively modest, potentially limiting statistical power to detect smaller effects, particularly interaction effects and associations involving less prominent AQ subscales. Second, autistic traits were assessed at a single time point using the standard AQ administration, precluding conclusions regarding their temporal stability or their relationship to symptom fluctuations over time. Third, OCD diagnoses and psychiatric comorbidities were established through routine clinical evaluation rather than structured diagnostic interviews, reflecting the naturalistic retrospective design of the study. Fourth, the retrospective design limits causal inference and relies on routinely collected clinical data. Finally, pharmacological treatments were not experimentally standardized, although all participants received guideline-based treatment within the same outpatient clinic under routine clinical care.

Despite these limitations, the present study has several important strengths. It represents one of the few longitudinal investigations examining dimensional autistic traits in adults with OCD using repeated clinician-rated symptom assessments. Additional strengths include the naturalistic clinical setting, longitudinal assessment of symptom severity at three time points, the application of complementary analytical approaches, and the examination of both overall autistic trait burden and specific AQ dimensions. The convergence of findings across linear mixed-effects models and RM-ANCOVA enhances confidence in the robustness of the observed associations.

In conclusion, elevated autistic traits were associated with consistently greater OCD symptom severity throughout treatment, with this relationship appearing to be particularly evident for compulsive symptoms. Exploratory analyses further suggested that social skills, rather than attention to detail, together with attention switching, were the autistic trait dimensions most strongly associated with symptom burden. Importantly, autistic traits did not alter the trajectory of treatment-related improvement, indicating that patients with higher autistic trait levels remained consistently more symptomatic across follow-up while achieving comparable relative benefit from standard pharmacological treatment. These findings support a dimensional perspective in which autistic traits contribute to the clinical expression of OCD without necessarily conferring poorer treatment responsiveness, and they highlight the need for prospective studies integrating social-cognitive, neuropsychological, and mechanistic assessments to clarify the pathways linking autistic traits to obsessive–compulsive symptomatology.

## Data Availability

The original contributions presented in the study are included in the article/**Supplementary Material**, Further inquiries can be directed to the corresponding author/s.
