# Supplementary material for: Autistic traits and obsessive–compulsive symptom severity: a retrospective six-month follow-up study
Source: Front Psychiatry. 2026 Sep 10;17:1940901. doi: 10.3389/fpsyt.2026.1940901 (PMC13601252; doi:10.3389/fpsyt.2026.1940901)
Supplement: Supplementary file 1 [file SupplementaryFile1.docx]

**Supplementary Material**

The supplementary material provides additional methodological and statistical information supporting the main findings of this study. It includes supplementary details regarding routine clinical assessment procedures, participant recruitment, Autism Spectrum Quotient (AQ) administration and interpretation, previous treatment characteristics, pharmacological treatment distribution during follow-up, and detailed statistical results from the primary and sensitivity analyses. All statistical analyses were performed using IBM SPSS Statistics version 25.0.

**Supplementary Methods**

**Routine Clinical Assessment**

Since 2023, obsessive–compulsive symptom severity has been routinely monitored in the Psychiatry Outpatient Clinic of Dinar State Hospital using the Yale–Brown Obsessive–Compulsive Scale (Y-BOCS) as part of standard clinical practice. Routine outpatient visits typically last approximately 20 minutes and include psychiatric evaluation, assessment of treatment response, medication adherence, adverse effects, psychiatric comorbidities according to DSM-5 clinical criteria, and clinician-administered Y-BOCS ratings. Clinical diagnoses were established through routine longitudinal psychiatric evaluation rather than structured research interviews.

**Participant Recruitment**

Between January and July 2025, consecutive patients who had completed at least six months of follow-up and met all eligibility criteria were invited to participate during a routine outpatient visit. After receiving information regarding the study procedures, eligible patients who provided written informed consent completed the Autism Spectrum Quotient (AQ). No additional structured diagnostic interviews or research assessments were performed. Longitudinal Y-BOCS data were retrospectively extracted from routine clinical records.

**Autism Spectrum Quotient (AQ)**

The Turkish version of the AQ has demonstrated acceptable psychometric properties, with a Cronbach's α coefficient of 0.64 for the total score and coefficients ranging from 0.32 to 0.52 across the five subscales (28). Although previous studies have proposed AQ thresholds (e.g., ≥26 as a screening threshold and ≥32 as suggestive of clinically significant autistic traits), the original developers emphasized that AQ scores should be interpreted as dimensional indicators rather than diagnostic criteria for autism spectrum disorder (27). Accordingly, AQ total and subscale scores were analyzed as continuous variables throughout the present study without classification according to predefined cut-off values.

**Supplementary Tables**

**Supplementary Table S1.** Distribution of Pharmacological Treatments During the Six-Month Follow-up

| **Treatment Type** | **n** | **%** | **Description** |
| --- | --- | --- | --- |
| Escitalopram monotherapy | 11 | 28.9 | SSRI monotherapy |
| Escitalopram + Aripiprazole | 8 | 21.1 | SSRI + antipsychotic augmentation |
| Clomipramine (± antipsychotic) | 7 | 18.4 | TCA ± antipsychotic augmentation |
| Venlafaxine (± antipsychotic) | 5 | 13.2 | SNRI ± antipsychotic augmentation |
| Paroxetine or Sertraline monotherapy | 2 | 5.3 | SSRI monotherapy |
| Other SSRI + Antipsychotic combinations | 5 | 13.2 | Mixed regimens within standard clinical protocols |

**Note:** Data represent the primary pharmacological regimen maintained during most of the six-month follow-up period. All treatments were prescribed according to contemporary clinical guidelines for obsessive–compulsive disorder. Percentages may not total 100 due to rounding.

**Supplementary Table S2.** **Previous Treatment Characteristics Before Study Inclusion**

| **Variable** | **n (%)** |
| --- | --- |
| Previous pharmacological treatment only | 19 (50.0) |
| Previous CBT and/or ERP only | 1 (2.6) |
| Previous pharmacological treatment + CBT/ERP | 1 (2.6) |
| No previous OCD treatment | 17 (44.7) |

**Note:** Previous treatment history refers to interventions received before the six-month follow-up period included in the present study. Pharmacological treatment history includes any documented antidepressant or augmentation therapy. CBT/ERP = cognitive-behavioral therapy with exposure and response prevention.

**Supplementary Table S3.** Linear Mixed-Effects Model Results for Y-BOCS Total, Obsession, and Compulsion Scores

| **Model** | **Fixed Effect** | **F (df)** | **p** | **AIC** | **BIC** |
| --- | --- | --- | --- | --- | --- |
| **Y-BOCS Total Severity** | **Time** | **20.824 (2, 47.557)** | **<.001** | **680.210** | **710.308** |
|  | **AQ Total** | **5.785 (1, 38.616)** | **.021** |  |  |
|  | Age | 1.038 (1, 37.958) | .315 |  |  |
|  | Sex | 0.010 (1, 37.958) | .921 |  |  |
|  | Time × AQ | 0.301 (2, 47.557) | .742 |  |  |
| **Y-BOCS Obsessions** | **Time** | **22.038 (2, 46.908)** | **<.001** | **518.025** | **548.123** |
|  | AQ Total | 2.544 (1, 38.747) | .119 |  |  |
|  | Age | 1.504 (1, 37.968) | .228 |  |  |
|  | Sex | 0.023 (1, 37.968) | .881 |  |  |
|  | Time × AQ | 0.338 (2, 46.908) | .715 |  |  |
| **Y-BOCS Compulsions** | **Time** | **16.710 (2, 47.834)** | **<.001** | **545.562** | **575.661** |
|  | **AQ Total** | **8.611 (1, 38.599)** | **.006** |  |  |
|  | Age | 0.490 (1, 37.969) | .488 |  |  |
|  | Sex | 0.112 (1, 37.969) | .740 |  |  |
|  | Time × AQ | 0.374 (2, 47.834) | .690 |  |  |

**Note:** Linear mixed-effects models were estimated using a random intercept for each participant and an unstructured covariance matrix for repeated measurements. Time, AQ total score, age, and sex were entered as fixed effects. The interaction between time and AQ total score was included to evaluate whether autistic traits influenced symptom trajectories over time. Models were estimated using maximum likelihood (ML). AIC = Akaike's Information Criterion; BIC = Bayesian Information Criterion.

**Supplementary Table S4.** Exploratory Repeated-Measures ANCOVA Results for Autism Spectrum Quotient Subscales

| **OCD Domain** | **AQ Subscales** | **F(1, 34)** | **p** | **η²ₚ** |
| --- | --- | --- | --- | --- |
| **Y-BOCS Total Severity** | Social Skills | 12.75 | .001 | .273 |
|  | Attention Switching | 9.34 | .004 | .215 |
|  | Communication | 2.47 | .125 | .068 |
|  | Imagination | 0.59 | .447 | .017 |
|  | Attention to Detail | 0.01 | .920 | .000 |
| **Y-BOCS Compulsions** | Social Skills | 11.55 | .002 | .254 |
|  | Attention Switching | 9.96 | .003 | .227 |
|  | Communication | 2.78 | .105 | .075 |
|  | Imagination | 0.02 | .878 | .001 |
|  | Attention to Detail | 0.01 | .939 | .000 |

**Note:** Each row represents a separate repeated-measures ANCOVA model including age and sex as covariates. AQ subscales were entered individually to avoid multicollinearity. Partial η² values of .01, .06, and .14 represent small, medium, and large effect sizes, respectively.
